# Molecular Phylogeny and Biogeography of *Petaurista* Inferred from the Cytochrome *b* Gene, with Implications for the Taxonomic Status of *P. caniceps*, *P*. *marica* and *P. sybilla*

**DOI:** 10.1371/journal.pone.0070461

**Published:** 2013-07-26

**Authors:** Song Li, Kai He, Fa-Hong Yu, Qi-Sen Yang

**Affiliations:** 1 State Key Laboratory of Genetic Resources and Evolution, Kunming Institute of Zoology, Chinese Academy of Sciences, Kunming, Yunnan, China; 2 Kunming Natural History Museum of Zoology, Kunming Institute of Zoology, Chinese Academy of Sciences, Kunming, Yunnan, China; 3 Department of Biological Science, University of Manitoba, Winnipeg, Manitoba, Canada; 4 ICBR, University of Florida, Gainesville, Florida, United States of America; 5 Key Laboratory of Zoological Systematics and Evolution, Institute of Zoology, Chinese Academy of Sciences, Beijing, China; BiK-F Biodiversity and Climate Research Center, Germany

## Abstract

The polymorphic genus *Petaurista* includes a group of diverse species and subspecies that are adapted for gliding and arboreal life. This morphological diversity has resulted in taxonomic discrepancies, and molecular phylogenetic studies have been limited by taxon sampling. To clarify this controversial taxonomy, we used the cytochrome *b* gene to reconstruct the phylogeny to obtain a more accurate picture of the evolutionary relationships, species differentiation and divergence pattern of *Petaurista*. The results revealed a significant inconsistency between taxonomic designations, phylogeny and genetic distances. When 6 recognized species were included, species delimitation revealed 15 putative species, a finding that warrants a comprehensive morphological diagnosis and a re-assessment of the species status. The validity of *P. caniceps* and *P. marica* was discussed. An estimation of the molecular divergence time demonstrated that the diversification and speciation of *Petaurista* began during the later Miocene and may have been affected by the uplifting of the Qinghai-Tibet plateau and subsequent climate change.

## Introduction

The giant flying squirrels, *Petaurista* Link, 1795, belong to the subfamily Sciurinae and are distributed from Pakistan and Nepal to East Asia, North Indochina and Southeast Asia [1]–[10]. This polymorphic genus includes a group of diverse species/subspecies that are adapted for gliding and arboreal life. The head and body lengths of these animals range widely from 305 mm to 585 mm, and the dorsal pelage exhibits a great variety of colors including yellowish gray, buffy gray, bright brown, chestnut and black [1], [10].

Within *Petaurista*, highly variable external morphology presents taxonomic difficulties, particularly for the trans-Himalayan taxa. At least 8 contradictory taxonomic hypotheses have been proposed based on dental and cranial characteristics and external morphology since 1940 (Table 1). In the latest taxonomic revision, 8 species were recognized [6]. Nonetheless, the number of recognized species has continuously changed from 5 to 31, and long-standing controversies remain regarding the taxonomic status of *P. albiventer*, *P. caniceps*, *P. hainana*, *P. marica*, *P. marica sybilla* and *P. yunanensis* (Table 1). For example, *P. caniceps, P. marica* and *P. sybilla* have been recognized as species, subspecies or synonyms in different revisions [2]–[10]. Notably, these taxonomic revisions were based on preliminary morphological comparison, and comprehensive morphological or morphometric analyses have not yet been performed.

**Table 1:** Taxonomic hypotheses of *Petaurista.*

Allen, 1940	Ellerman,1940^a^	Ellerman &Morrison-Scott, 1950	Corbet &Hill, 1992	Zhang et al.,1997	Nowak, 1999	Wang, 2003	Hoffman(1993);Thoringtonet al., 2005
*P. petaurista*	*P. petaurista*	*P. petaurista*	*P. petaurista*	*P. petaurista*	*P. petaurista*	*P. petaurista*	*P. petaurista*
*P. alborufus*	*P. alborufus*	*P. alborufus*	*P. alborufus*	*P. alborufus*	*P. alborufus*	*P. alborufus*	*P. alborufus*
*P. yunanensis*	*P. yunnanensis*			*P. yunanensis*			
*P. hainanus*	*P. hainana*			*P. hainana*			
	*P. philippensis*		*P. philippensis*	*P. philippensis*	*P. philippensis*	*P. philippensis*	*P. philippensis*
	*P. albiventer*					*P. albiventer*	
*P. xanthotis*	*P. xanthotis*		*P. xanthotis*	*P. xanthotis*	*P. xanthotis*	*P. xanthotis*	*P. xanthotis*
	*P. leucogenys*	*P. leucogenys*			*P. leucogenys*		*P. leucogenys*
	*P. magnificus*	*P. magnificus*	*P. magnificus*	*P. magnificus*	*P. magnificus*	*P. magnificus*	*P. magnificus*
			*P. nobilis*		*P. nobilis*		*P. nobilis*
	*P. grandis*						
	*P. lena*						
	*P. elegans*	*P. elegans*	*P. elegans*	*P. elegans*	*P. elegans*	*P. elegans*	*P. elegans*
				*P. marica*			
	*P. caniceps*		*P. caniceps*		*P. caniceps*	*P. caniceps*	
			*P. sybilla*		*P. sybilla*	*P. sybilla*	
	*P. pectoralis*			*P. pectoralis*			
	*P. watasei*					*P. watasei*	
*P. punctatus*	*P. punctatus*						
*P. calrkei*	*P. clarkei*						

a Ellerman (1940) recognized 31 species, 14 of which (P. cineraceus, P. lylei, P. mergulus, P. annamensis, P. candidulus, P. taylori, P. fulvinus, P. inornatus, P. birrelli, P. gorkhali, P. melanopterus, P. sulcatus, P. rubicundus and P. filchnerinae) have not been recognized as valid Petaurista species by any other researcher.

**Figure 1:**
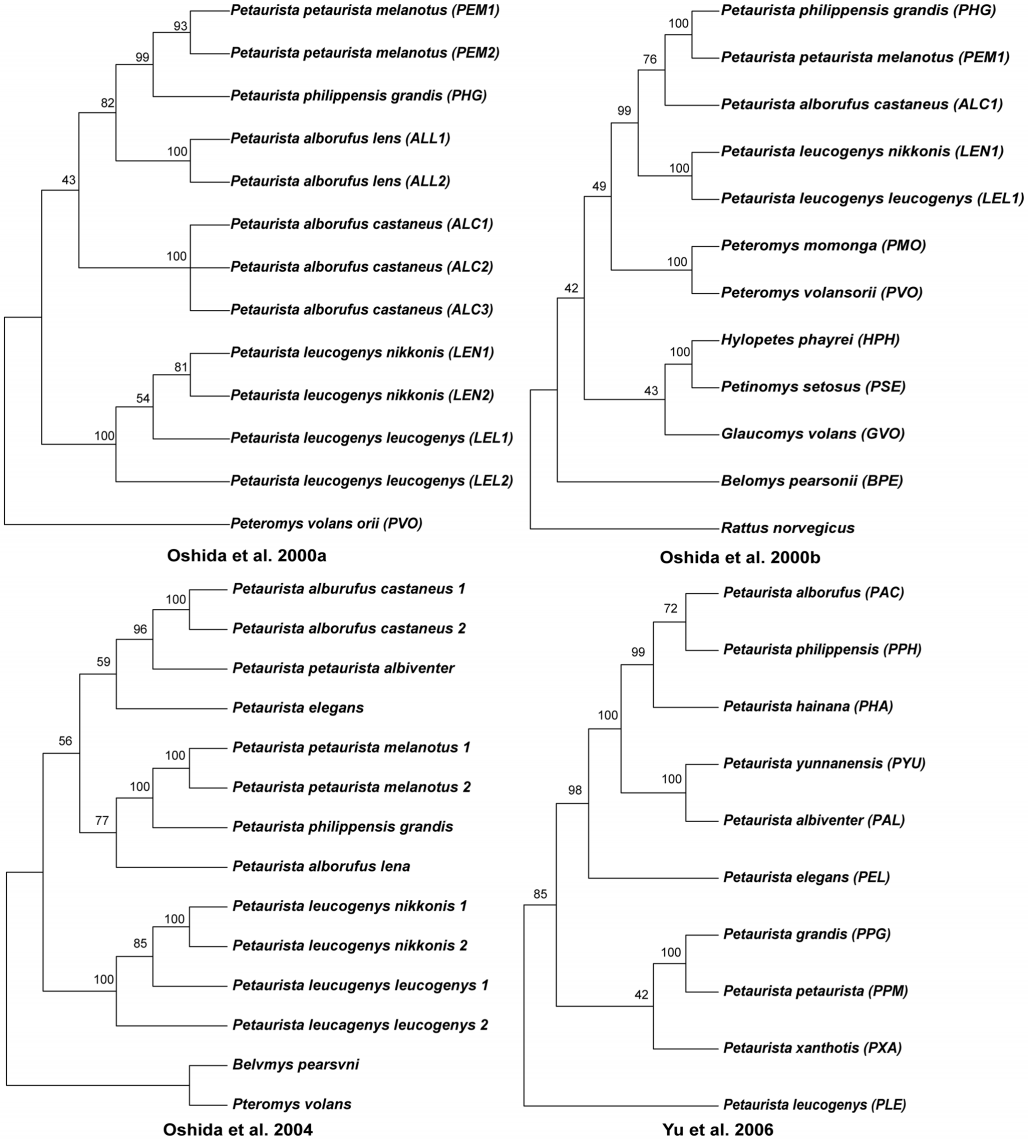
Phylogenies of *Petaurista* species based on cyt *b* (Oshida et al., 2000a, 2000b, 2004; Yu et al., 2006).

Only recently have molecular phylogenies of *Petaurista* been proposed, all of which are based on cytochrome *b* (cyt *b*) [11]–[16]. However, due to the disparate sampling of taxa, no broad picture of the phylogeny is available (Figure 1). In addition, no genetic information has been published for *P. caniceps, P. marica* and *P. sybilla*.

In this study, to better understand the phylogeny and evolutionary history of *Petaurista*, we obtained the cyt *b* sequences of *P. caniceps*, *P. philippensis*, *P. yunanensis*, *P. marica* and *P. sybilla*. With additional sequences from GenBank, 6 of 8 species recognized by Thorington et al. (2005) were sampled, enabling us to develop a broad picture of *Petaurista* evolution and to investigate the validity of some debatable *Petaurista* species, such as *P. sybilla* and *P. caniceps*. Furthermore, we used Bayesian relaxed molecular clock approaches and fossil data to analyze the correlation between the evolutionary history of *Petaurista* and climate change.

## Materials and Methods

### Ethics Statement

All samples used in this study were obtained from specimens deposited in the Kunming Natural History Museum of Zoology (KNHMZ) at the Kunming Institute of Zoology (KIZ) of the Chinese Academy of Sciences (CAS). Our sampling did not violate any law, rule or regulation in China and thus required no ethical or institutional approval. Additionally, we obtained permission from the KNHMZ and the KIZ of the CAS to use the samples in our study (no permit number).

### Sampling

In total, 10 specimens of *Petaurista* were collected and deposited in KNHMZ, KIZ. Additionally, 25 cyt *b* sequences of *Belomys, Petaurista* and *Pteromys* were obtained from GenBank. Thus, our sampling included 6 *Petaurista* species recognized by Thorington and Hoffmann [6] (Table 2).

**Table 2:** Taxon and sequences used in this study.

Taxon	Accession No.	Sample Locality
*Pteromys volans*	AB097683	Japan
*Belomys pearsonii*	AB126245	Taiwan, China
*Petaurista leucogenys* #	AB092616	Fukuoka, Japan
	AB092617	Ehime, Japan
	AB092618	Wakayama, Japan
	AB092619	Nagano, Japan
*Petaurista xanthotis* #	DQ072111	Gansu, China*
*Petaurista caniceps*	JQ928705*	Mile, Yunnan, China
	JQ928704*	Jingdong, Yunnan, China
	JQ928703*	Gongshang, Yunnan, China
*Petaurista petaurista* #	AB092608	Laos
	AB092609	South China
	AB023909	South China
	AB023908	Laos
*Petaurista grandis*	AB092611	Nantou, Taiwan, China
	AB023907	Nantou, Taiwan, China
*Petaurista philippensis* #	DQ072107	Yunnan, China
	JQ928697*	Shiping, Yunnan, China
*Petaurista alborufus* #	AB092613	South China
	AB092614	South China
*Petaurista lena*	AB023901	Nantou, Taiwan, China
	AB023902	Hualien, Taiwan, China
	AB092615	Nantou, Taiwan, China
*Petaurista hainana*	DQ072108	Hainan, China
*Petaurista albiventer*	DQ072109	Pakistan
	AB092612	Ayubia National Park, Pakistan
*Petaurista yunanensis*	JQ928701*	Yunlong, Yunnan, China
	JQ928702*	Gongshan, Yunnan, China
	DQ072110	Yunnan, China
*Petaurista elegans* #	AB092610	Jambi, Indonesia
	AB047380	-
*Petaurista marica*	JQ928700*	Lvchun, Yunnan, China
	JQ928696*	Jinping, Yunnan, China
*Petaurista sybilla*	JQ928699*	Gongshan, Yunnan, China
	JQ928698*	Gongshan, Yunnan, China

* Novel data collected in this study.

# Species recognized by Thorington and Hoffmann (2005).

### DNA Preparation and Sequencing

The samples used in this study were muscle tissues preserved in 95% ethanol or pedal skin specimens. Before DNA extraction, pedal skins were treated in a series of 48-hour washes in 90, 70, 50, 30 and 10% ethanol, followed by successive 24-hour immersions in phosphate-buffered saline (PBS). Total DNA was extracted using a Tissue DNA Kit (BioTeke Corporation, Beijing, China), according to the manufacturer’s protocols. Cyt *b* sequences were amplified using a set of primer pairs, including L14724, L14979, H15149, H15915 [17], [18], L15306, H15347, and H15603 [12], as well as 2 other primers that were designed in this study: L15460 (5′-CTC ATA ATC CTA GTC CTA T T-3′) and L15550 (5′-ACA TTA AAC CAG AAT GAT ACT TCC TAT-3′). The 50-µl polymerase chain reaction (PCR) mixture contained 25 µl of 2×Power Taq PCR MasterMix (BioTeke Corp.), 2 µl (10 ng) of genomic DNA and 2 µl of each primer (10 pmol). PCR amplification was performed using the following program: 5 min at 94°C, followed by 35 cycles of 1 min at 94°C, 1 min and 10 s at 48–54°C, then 1 min at 72°C and a post-extension step for 10 min at 72°C. The PCR products were purified and sequenced using the BigDye Terminator Cycle kit 3.1 on an ABI 3730xl sequencer. All experiments were performed in a biological safety cabinet (Air Tech SW-CJ-1FD, Suzhou Antai Air Tech Co. Ltd.). Negative controls were used in all DNA extraction and PCR amplifications to control for potential contamination.

### Sequence Alignment

Nucleotide sequences were proofread using SeqMan (DNAstar Inc., Madison, WI) and were aligned using Clustal W [19]. Quantitative pairwise comparisons between *Petaurista* putative species were performed, and the average genetic distances between phylogenetic clades were calculated using Kimura’s (1980) 2-parameter (K2P) method in MEGA 5.0 [20]. To test the homogeneity of base frequencies across taxa, PAUP* 4.0b10 [21] was used to conduct a chi-squared test.

### Phylogenetic Analysis

To elucidate the phylogenetic relationships among the *Petaurista* species, phylogenetic analyses were performed to assess maximum likelihood (ML) with GARLI v2.0 [22] and Bayesian inference (BI) using MrBayes v3.2.1 [23]. The cyt *b* data used in this study were partitioned according to the codon position for both ML and BI analyses. The best-fit evolutionary model of each codon position was calculated using jModeltest v2.1 [24] and determined using the Bayesian information criterion (BIC) because of its high accuracy and precision [25]. The models used for the 1^st^, 2^nd^ and 3^rd^ codon positions were SYM+G, HYK+G and GTR+G, respectively.

ML tree calculation was performed using a random starting tree, 5 replicate searches and 5 million generations for each replicate, and were sampled every 1,000 generations to estimate the best tree. The bootstrap support (BS) was assessed based on 1,000 bootstrap replicates. PAUP* 4.0b10 [21] was used to generate the strict consensus tree.

Partitioned Bayesian analyses were executed using a random starting tree and the program’s default distributions for model parameters. The analyses were repeated twice, and each analysis included 30 million generations. The results were sampled every 3,000 generations. Convergences were assessed by calculating the effective sample sizes (ESSs) using Tracer v1.5 [26]. Conservatively, the first 25% of the sampled trees were discarded as “burn in”, and the remaining 75% of the sampled trees were used to calculate the Bayesian posterior probabilities (PP). Nineteen alternative phylogenetic hypotheses were also tested using CONSEL v0.2 [27] and PAUP4.0b10 by calculating the p-value in the approximately unbiased (AU) [28], Kishino–Hasegawa (KH) [29] and Shimodaira–Hasegawa (SH) [30] tests. Selection bias results from comparing many trees; in this case, the AU test is less biased regarding tree selection [28].

### Divergence Time Estimation and Species Delimitation

The divergence times were estimated using the uncorrelated relaxed molecular clock approach [31] implemented in BEAST v1.7.4 [32]. Fourteen additional Sciurid taxa (the GenBank accession numbers are shown in the tree) were included as outgroups. Two calibration ages were treated as lognormal distributions with soft boundaries [33] and were defined based on fossil records in the Paleobiology Database (http://paleodb.org) and the NOW (New and Old Worlds) database of fossil mammals [34]. The oldest fossil squirrel (*Douglassciurus jeffersoni*) is known from the late Eocene (37.2–33.9 million years ago [Ma]). A previous study demonstrated that this calibration should be applied to a crown group of squirrels [35]. Thus, we used this fossil to calibrate the most recent common ancestor (MRCA) of living squirrels. The fossil sites were dated to 37.86–35.75 Ma, and changing the calibration date to between 37.8 and 35 Ma has an insignificant effect (see [35] and references therein). We used a lognormal distribution such that the earliest possible sample age was 33.9 Ma and the older 95% credible interval (CI) included 37.2 Ma (offset = 33.9, mean = 1.05, and standard deviation = 1.0). Note that we used a younger lower boundary because the cyt *b* sequences of *Ratufa* and *Sciurillus,* which represent the basal taxa of squirrels, were not included (or available) [35]. The fossil records of *P. petaurista* appeared in the strata of the middle to late Pleistocene (1.3–0.6 Ma [34], [36]). Thus, we set the earliest possible age to 1.3 Ma and the older 95% CI to 2.4 Ma at the Pliocene/Pleistocene boundary, when the climate shifted toward cooler and drier conditions (offset = 1.3, mean = 0.35, and standard deviation = 1.0) [37]. The substitution models used for each codon position were the same as those used in the MrBayes analyses. Each BEAST analysis included a randomly generated starting tree, an uncorrelated lognormal relaxed molecular clock, a birth-death model for the tree, and 10 million generations that were sampled every 1,000 generations. Tracer 1.5 [26] was used to confirm that each independent analysis had reached stationary states (i.e., ESSs >200).

Based on the time-calibrated tree calculated using BEAST, the number of putative species was identified using the single threshold GMYC model [38]. This method used maximum-likelihood statistics and divergence times in a tree to identify the split point from the species to the population level. In some cases, this method performed very well (errors less than 25%) [39], and the temporal pattern of diversification was visualized using lineages-through-time (LTT) plots. We calculated Pybus and Harvey’s γ to determine whether diversification occurred earlier (γ<0) or later (γ>0) [40]. These analyses were implemented using the APE v3.0, Laser v2.3 and SPLITS v2.1 packages for the R statistical environment [41], [42].

## Results

### Gene Sequences

We analyzed 35 cyt *b* sequences (1068–1140 bp), including 6 of 8 *Petaurista* species recognized by Thorington and Hoffmann [6] (Table 2). The sequences of *P. caniceps, P. marica* and *P. marica* are novel data (Table 2). The average nucleotide composition of the cyt *b* genes was 28.1% A, 29.2% T, 12.8% G and 29.9% C. The sequence alignment included 449 variable sites along with 386 parsimony informative sites (33.9% of the entire sequence). Analysis of the base composition (P = 1.0, df = 96, Chi-Squared = 48.77) indicated homogeneity among the taxa. The K2P distances between pairs of species are listed in Table 3. The pairwise distance values among *P. caniceps*, *P. elegans*, *P. marica* and *P. sybilla* were between 4.80 and 16.47% (Table 3).

**Table 3:** Genetic differences of *Petaurista* taxa based on pairwise comparisons of complete cytochrome *b* gene sequences (1,140 bp).

		1	2	3	4	5	6	7	8	9	10	11	12	13	14
1	*P. elegans*		2.45	2.45	8.92	8.25	7.92	7.59	6.14	7.43	5.66	7.48	7.26	5.82	6.94
2	*P. marica*	10.49		1.43	9.09	7.76	8.08	8.42	6.30	7.59	5.82	8.08	7.43	6.30	7.10
3	*P. sybilla*	11.53	4.80		9.09	7.76	8.08	8.42	6.30	7.59	5.82	8.08	7.43	6.30	7.10
4	*P. caniceps*	16.47	15.18	15.16		5.98	4.42	2.11	5.19	1.96	5.98	3.40	6.94	6.46	6.62
5	*P. leucogenys*	17.09	15.65	16.19	15.00		5.03	5.98	4.57	5.82	5.35	5.03	6.94	6.30	6.14
6	*P. xanthotis*	15.47	14.66	15.65	14.29	11.79		4.41	4.26	6.26	5.03	4.11	6.62	4.88	5.66
7	*P. petaurista*	15.58	14.65	14.84	10.21	14.64	12.15		5.19	1.00	5.98	3.20	6.30	6.14	6.62
8	*P. philippensis*	16.85	15.64	16.71	14.29	13.99	15.47	13.96		5.03	1.00	4.26	2.45	2.60	2.75
9	*P. grandis*	15.04	13.64	14.15	10.72	14.67	12.02	3.99	13.58		5.19	3.05	6.14	6.30	6.46
10	*P. alborufus*	16.82	15.73	17.66	15.91	14.34	15.26	14.00	6.59	13.83		5.03	2.60	2.75	2.90
11	*P. lena*	16.57	14.71	16.14	12.99	14.95	14.21	12.19	15.58	13.35	16.87		6.62	5.50	5.66
12	*P. hainana*	16.24	15.66	16.93	15.04	13.17	13.43	12.73	6.09	13.19	7.69	15.55		4.26	4.41
13	*P. albiventer*	16.89	14.36	16.23	15.65	16.09	14.35	14.62	10.69	14.41	11.51	16.02	10.88		1.57
14	*P. yunanensis*	16.39	15.11	16.70	15.17	16.37	15.98	14.44	9.94	14.53	11.19	15.93	10.35	6.83	

Data above the diagonal represent the transversional percentage differences of the 3rd codon position of sequences among taxa. Data below the diagonal are the percentage differences of sequences among taxa.

### Phylogenetic Relationships among *Petaurista*

**Figure 2:**
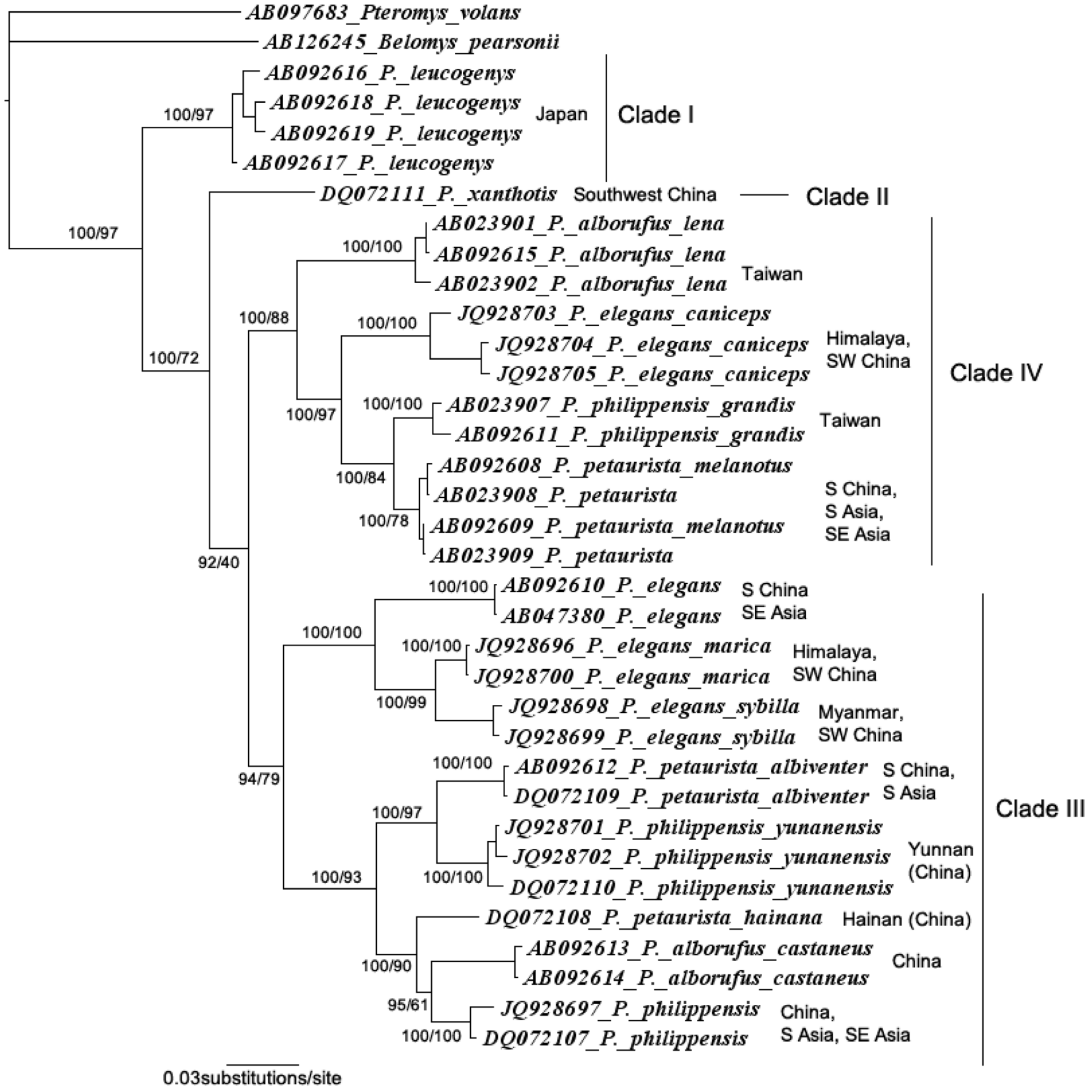
Phylogenetic relationships of *Petaurista* constructed based on 1,068–1,140 bp of the cyt *b* gene using the ML method and BI. Numbers above the branches are Bayesian posterior probabilities/likelihood bootstrap values.

Phylogenetic reconstructions using ML and BI generated the same tree topology, with overall strong supports (i.e., PP>95% and BS >70%; [43], [44]) for most but not all relationships (Figure 2). With *Belomys pearsonii* and *Pteromys volans* as outgroup taxa, *Petaurista* was consistently supported as a monophyletic clade (PP = 100% and BS = 97%), in which 4 major phylogroups were recovered (clades I, II, III and IV). Clade I, which is represented by a single species, *P. leucogenys,* occupied a basal position within the genus (PP = 100% and BS = 72%). Clade II (represented by *P. xanthotis*) and clades III+IV are sister groups, but the sister relationship between clades III and IV was not supported by bootstrap replicates or Bayesian probabilities (PP = 92% and BS = 40%), indicating that the relationship was not stable. Further AU, KH and SH tests found that 11 alternative phylogenetic scenarios could not be rejected by at least 1 test, and 4 could not be rejected by all 3 tests (P>0.05; Table 4). Thus, the relationships among the 4 clades remained ambiguous. Even so, *P. caniceps* represented a distinct lineage, which is the sister group of *P. petaurista*+*P. grandis* (PP = 100% and BS = 97%). *P. marica* and *P. sybilla* were supported as sister taxa (PP = 100% and BS = 99%), and the K2P distance between them was 4.80%.

**Table 4:** AU, KH and SH tests.

Scenarios	Tree length	Δl nL	AU	KH	SH
ML&BI topology	1290	-	**0.811**	**0.683**	**0.967**
(I,(II,III,IV))	1300	10.28	0.002	0.04	**0.476**
(I,II,(III,IV))	1308	23.23	<0.001	0.005	**0.079**
(I,(III,(II,IV)))	1292	3.75	**0.511**	**0.317**	**0.783**
(I,(IV,(II,III)))	1294	7.28	**0.176**	**0.141**	**0.607**
(II,(I, III,IV))	1311	33.73	0.001	0.001	0.003
(II,(I,(III,IV)))	1301	21.75	0.004	0.011	0.11
(II,(III,(I,IV)))	1304	28.88	0.003	0.01	0.025
(II,(IV,(I,III)))	1304	28.88	0.003	0.01	0.025
(III,(I,II,IV))	1309	25.21	<0.001	0.029	**0.050**
(III,(I,(II,IV)))	1293	12.54	**0.232**	**0.18**	**0.398**
(III,(II,(I,IV)))	1300	21.09	0.048	**0.069**	**0.125**
(III,(IV,(I,II)))	1297	11.10	**0.307**	**0.19**	**0.443**
(IV,(I,II,III))	1316	36.51	0.004	0.001	0.001
(IV,(I,(II,III)))	1308	31.50	<0.001	0.004	0.009
(IV,(II,(I,III)))	1310	32.92	<0.001	0.003	0.006
(IV,(III,(I,II)))	1301	17.46	0.015	**0.071**	**0.221**
((I,II),(III,IV))	1299	15.49	0.048	**0.078**	**0.283**
((I,III),(II,IV))	1298	18.62	0.018	**0.072**	**0.185**
((I,IV),(II,III))	1305	28.64	0.012	0.011	0.027

**Figure 3:**
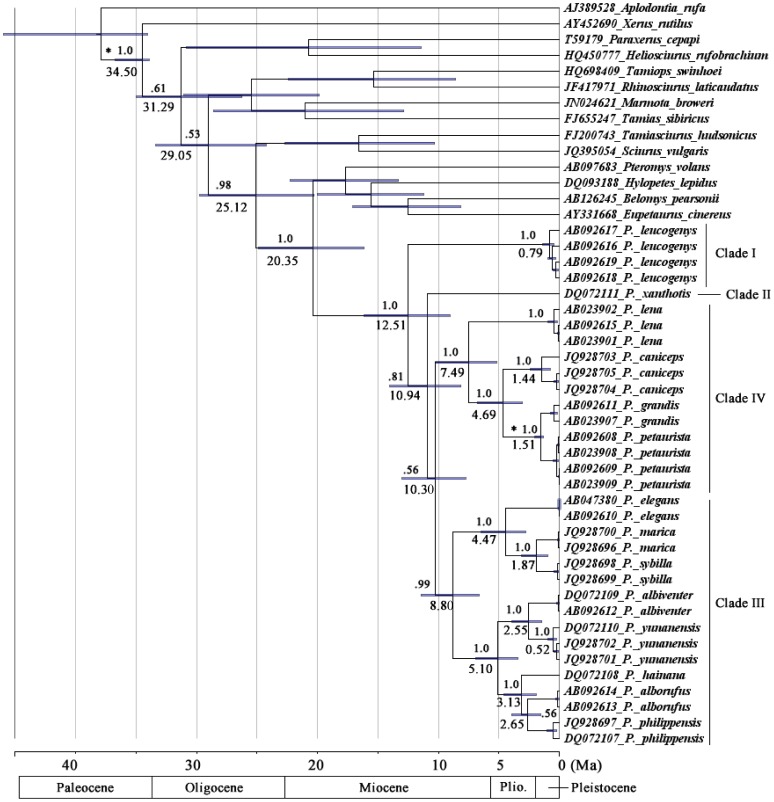
Chronogram of *Petaurista* from the partitioned Bayesian analysis using a relaxed molecular clock. Branch lengths represent time. Black dots represent nodes; the age of these nodes was calibrated based on fossil records.

Clade numbers are represented in Figures 2 and 3.

### Molecular Divergence Dating, Species Delimitation and Species Diversification

**Figure 4:**
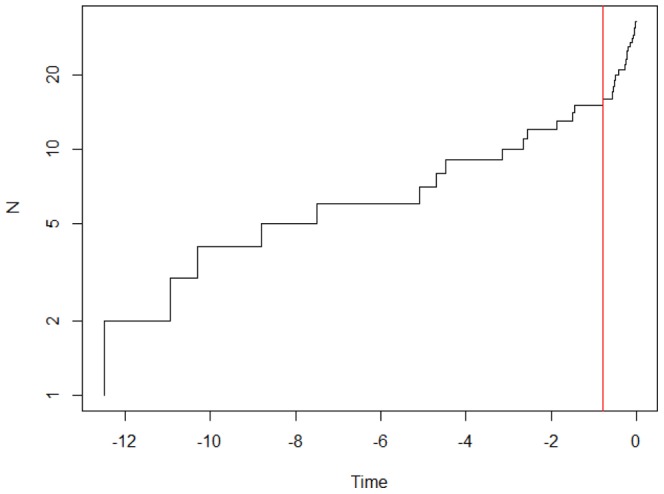
The diversification rate and species delimitation analyses.

BEAST analyses recovered the same topology as GARLI and MrBayes analyses (Figure 2). The MRCA of the genus existed at approximately 12.51 Ma (95% CI = 16.16–9.04) (Figure 3). The divergences among clades II - IV occurred approximately between 10.94 and 10.30 Ma (95%CI = 14.03–7.75). Note that because the relationships among the 4 clades are not stable, the divergence times and the results of the LTT plots and the Pybus and Harvey’s tests should be treated with caution. The early splits within clades III and IV occurred at 8.80 and 7.49 Ma, respectively (95%CI = 11.45–5.17). The results also revealed that the divergence of *P. caniceps* and *P. grandis+petaurista*, *P. elegans* and *P. sybilla*+*marica* as well as *P. albiventer+petaurista+yunanensis* and *P. alborufus+hainana+philippensis* occurred almost simultaneously at approximately 5.10–4.47 Ma (95%CI = 6.95–2.78). *P. marica* and *P. sybilla* split at approximately 1.87 Ma (95%CI = 3.12–0.97) (Figure 3). The LTT analysis demonstrated high rates of lineage accumulation at the early Pliocene (approximately 5–4 Ma) and late Pleistocene (approximately since 0.8 Ma) (Figure 4). Species delimitation analyses revealed 15 lineages as putative species; lineages that diverged older than 0.79 Ma were identified as potential species (Figure 3). Thus, despite the 6 species recognized by Thorington et al., 2005, *P. albiventer, P. hainana, P. lena, P. marica, P. sybilla* and *P. yunnanensis* were also recognized as potential species, and *P. caniceps* was recognized as 2 potential species. The Pybus and Harvey’s γ-value from our tree was −1.02 (P = 0.15). Thus, the pure-birth model was not significantly rejected, and does not support early-burst or late-burst/high early extinction.

## Discussion

### Phylogenetic Relationships among *Petaurista*

Although more sequences and species were included in our phylogenetic analyses, the topology is largely congruent with all previous hypotheses; that is, no conflicting relationship with high BS or PP was observed. Unfortunately, alternative phylogenetic scenarios could not be rejected statistically (Table 4); therefore, the relationships among the 4 major lineages, including the basal position of the genus, remain obscure. Indeed, the values of the log-likelihood (-lnL) of several alternative phylogenies are very close to that of the ML/BI topology (Table 4). The unresolved nature of the phylogeny might be attributed to insufficient phylogenetic information in the cyt *b* sequences. Other potential reasons include a rapid radiation. In the time-calibrated tree, the branches representing the 4 clades are short, and the divergences may have occurred within 2 million years (Figure 3). Regardless of the reason, more robust data, such as multiple unlinked nuclear genes, are required to fully resolve the relationships.

### Taxonomic Implications

Although we were not able to fully resolve the relationships, the 4 major clades and 15 putative species recognized in our analyses enable us to discuss the taxonomy of *Petaurista* at a preliminary level. We note that our species delimitation analysis was based on a single gene and a very simple hypothesis and relied on a genetic species concept [45], [46]. Therefore, these putative species represent only a crude estimate rather than a fully described model. To better understand the taxonomic status of these putative species, further investigation using multiple unlinked genes, comprehensive morphological and/or morphometric analyses, karyotypic studies and ecological and reproductive studies are warranted. Even so, the putative species recognized appear to be congruent with the previous taxonomic hypotheses (Table 1) and have been implied in phylogenetic studies [11], [12], [13]. The species status of *P. albiventer, P. grandis*, *P. hainana, P. lena* and *P. yunanensis* have been discussed by Oshida et al. [11], [12] and Yu et al. [13], and we will focus on the taxonomic status of *P. marica*, *P. caniceps* and *P. sybilla* herein.

*P. caniceps* was first recognized as *Sciuropterus caniceps* in 1842 [47]. The following taxonomic rearrangements appear to be author-dependent [2]–[6], [8]. In the present study, the distinct phylogenetic position and strikingly large genetic distances indicate that this species should be considered valid. In addition, *P. caniceps* is morphologically distinguishable from all other *Petaurista* by the absence of any unique white speckling over the back and a grey forehead. *P. caniceps* is sympatrically distributed with *P. marica* in southwestern China [5]. It is noteworthy that the species from western and middle Yunnan, China are also genetically distinguishable and were identified as 2 putative species in species delimitation analyses. Examination of the morphological differences among populations is warranted.

**Figure 5:**
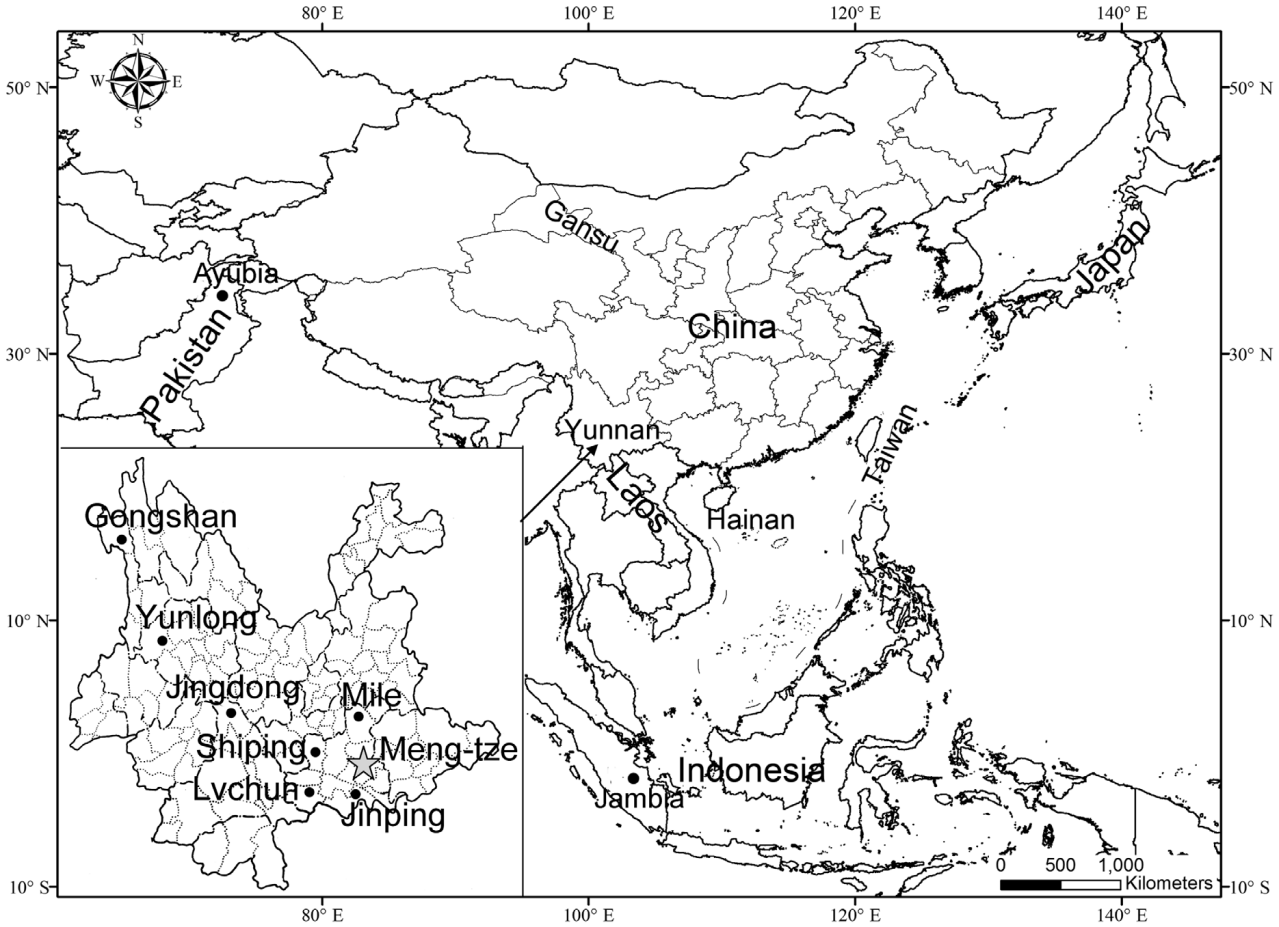
Sampling localities of *Petaurista* used in this study. An asterisk indicates the type locality of *P. marica*.

*P. marica* was first described by Thomas (1912) based on specimens from Yunnan (most likely near Mong-tze), China [48], and *P. sybilla* was named by Thomas and Wroughton in 1916 [49]. Since then, the taxonomic status of these species has been author dependent [2]–[6], [8]. In this study, *P. marica* is represented by 2 specimens from locations in Lvchun and Jinpin (Table 2) that are very close to its type locality (Figure 5); *P. sybilla* is represented by 2 samples from western Yunnan. The results suggest that *P. marica* and *P. sybilla* may have diverged from *P. elegans* 4.47 Ma (95%CI = 6.46–2.78) and that the former 2 taxa split during the early to middle Pleistocene (3.12–0.97 Ma). The results justify a re-assessment of these 2 taxa and call for comprehensive morphological diagnoses.

### Correlation between *Petaurista* Evolution and Climate Change

The higher-level phylogenetic relationships of *Petaurista* were not fully resolved and characterized by relatively short branches. Therefore, we assumed the diversification among the 4 major lineages to have occurred at approximately 12.51–7.49 Ma (95%CI = 16.16–5.17) and may be associated with episodes of global cooling since the middle Miocene (from 15 Ma) as well as the accelerated uplift of the Qinghai-Tibet Plateau (at approximately 10–8 Ma) [50]–[52]. The uplift of the plateau also strengthened the East Asia monsoon and increased the aridity of the dry seasons [51]. The climate change and consequent habitat turnover could have led to fragmentation of the *Petaurista* distribution. This suggestion is based on the observed short branches but is not supported by the Pybus and Harvey's test results. Nonetheless, rapid diversification was also observed at approximately 12–10 Ma among tree squirrel genera on the Sunda Shelf islands and has been connected to climate change and the subsequent drop in sea levels [35]. Most of the diversification among species occurred from the early Pliocene to the early Pleistocene (5–2 Ma), a finding that may be related to global cooling and desiccation, particularly around the Miocene/Pliocene boundary and during Pleistocene climate fluctuations [53]–[56]. However, these correlations require stronger evidence and should be tested in other East Asian taxa.
